# The intracellular detection of MIP-1beta enhances the capacity to detect IFN-gamma mediated HIV-1-specific CD8 T-cell responses in a flow cytometric setting providing a sensitive alternative to the ELISPOT

**DOI:** 10.1186/1742-6405-5-22

**Published:** 2008-10-06

**Authors:** Sarah Kutscher, Claudia J Dembek, Simone Allgayer, Silvia Heltai, Birgit Stadlbauer, Priscilla Biswas, Silvia Nozza, Giuseppe Tambussi, Johannes R Bogner, Hans J Stellbrink, Frank D Goebel, Paolo Lusso, Marco Tinelli, Guido Poli, Volker Erfle, Heike Pohla, Mauro Malnati, Antonio Cosma

**Affiliations:** 1 Institute of Virology, Helmholtz Zentrum München, German Research Center for Environmental Health, 85764 Neuherberg, Germany; 2 Clinical cooperation group "Immune monitoring", Helmholtz Zentrum München, German Research Center for Environmental Health, 85764 Neuherberg, Germany; 3 Institute of Virology, Technical University, 81675 Munich, Germany; 4 Human Virology Unit, San Raffaele Scientific Institute, 20132 Milan, Italy; 5 AIDS Immunopathogenesis Unit, San Raffaele Scientific Institute, 20132 Milan, Italy; 6 Laboratory of Clinical Immunology, San Raffaele Scientific Institute, 20132 Milan, Italy; 7 Department of Infectious Diseases, San Raffaele Scientific Institute, 20132 Milan, Italy; 8 Laboratory for Tumor Immunoloy, LIFE-Zentrum, Ludwig-Maximilians-Universität München, 81377 Munich, Germany; 9 Department of Infectious Diseases, Med. Poliklinik, University Hospital of Munich, 80336 Munich, Germany; 10 IPM Study Center, 20146 Hamburg, Germany; 11 Division of Infectious and Tropical Diseases, Hospital of Lodi, 26866 Lodi, Italy; 12 Vita-Salute San Raffaele University, School of Medicine, 20132, Milano, Italy

## Abstract

**Background:**

T-cell mediated immunity likely plays an important role in controlling HIV-1 infection and progression to AIDS. Several candidate vaccines against HIV-1 aim at stimulating cellular immune responses, either alone or together with the induction of neutralizing antibodies, and assays able to measure CD8 and CD4 T-cell responses need to be implemented. At present, the IFN-γ-based ELISPOT assay is considered the gold standard and it is broadly preferred as primary assay for detection of antigen-specific T-cell responses in vaccine trials. However, in spite of its high sensitivity, the measurement of the sole IFN-γ production provides limited information on the quality of the immune response. On the other hand, the introduction of polychromatic flow-cytometry-based assays such as the intracellular cytokine staining (ICS) strongly improved the capacity to detect several markers on a single cell level.

**Results:**

The cumulative analysis of 275 samples from 31 different HIV-1 infected individuals using an ICS staining procedure optimized by our laboratories revealed that, following antigenic stimulation, IFN-γ producing T-cells were also producing MIP-1β whereas T-cells characterized by the sole production of IFN-γ were rare. Since the analysis of the combination of two functions decreases the background and the measurement of the IFN-γ+ MIP-1β+ T-cells was equivalent to the measurement of the total IFN-γ+ T-cells, we adopted the IFN-γ+ MIP-1β+ data analysis system to evaluate IFN-γ-based, antigen-specific T-cell responses. Comparison of our ICS assay with ELISPOT assays performed in two different experienced laboratories demonstrated that the IFN-γ+ MIP-1β+ data analysis system increased the sensitivity of the ICS up to levels comparable to the sensitivity of the ELISPOT assay.

**Conclusion:**

The IFN-γ+ MIP-1β+ data evaluation system provides a clear advantage for the detection of low magnitude HIV-1-specific responses. These results are important to guide the choice for suitable highly sensitive immune assays and to build reagent panels able to accurately characterize the phenotype and function of responding T-cells. More importantly, the ICS assay can be used as primary assay to evaluate HIV-1-specific responses without losing sensitivity in comparison to the ELISPOT assay.

## Background

Vaccine development has become more complex in the last decades, pursuing new strategies for stimulating immune responses against infectious agents of viral, bacterial or parasitic origin as well as against cancer. A striking example is the long-winded search for an effective HIV-1 vaccine that would be crucial, together with antiretroviral therapy, to limit and possibly stop the worldwide AIDS pandemic. Several candidate HIV-1 vaccines that aim to stimulate cellular immune responses have been tested in phase I and II clinical trials [1-3]. An accurate evaluation of the cellular immune response will be key to select vaccine candidates for successive phase III clinical trials. Therefore, methods that qualify and quantify antigen-specific, functional T cells in a precise, sensitive, and robust way will be essential. At present, the standard assays that are commonly used for this purpose are IFN-γ ELISPOT, HLA class I and class II multimer staining and ICS. The ELISPOT assay is currently considered the gold standard in vaccine trials due to its sensitivity and extensive standardization and validation [4-7]. In fact, several reports demonstrated that the ELISPOT assay is more sensitive in detecting weak responses when compared to the ICS assay [8-11], a feature that represents an important advantage for the detection and measurement of the immune response in vaccine trials [12]. The most commonly used ELISPOT assay measures IFN-γ secretion by total PBMC stimulated by specific antigens. Albeit ELISPOT assays being able to measure the secretion of two different cytokines have been recently established [13], it is unlikely that future development will increase the simultaneous measurement of cytokines for this kind of assays. On the other hand, the introduction of new reagents, instruments and software, strongly improved the capacity of flow cytometry based assays such ICS and multimer staining to simultaneously measure several parameters in the same sample [14-16]. However, between ICS and multimer staining, the former seems to be more suited to be employed in vaccine trials since it does not require previous HLA typing and *a priori *knowledge of specific epitopes [17,18]. Hence, it is generally accepted that ICS provides more information regarding the quality of the immune response whereas ELISPOT grants a high capacity of detecting low magnitude responses, while multimer staining is the method of choice for a detailed analysis of the immune response in a selected and limited number of samples.

In spite of an intense activity in the development and testing of new vaccines against HIV-1, clear immunological correlates of protection do not still exist although there is strong evidence that CD4 and CD8 T-cells play a role in the control of viral replication [19]. However, neither the magnitude of the immune response (measured as production of IFN-γ) nor the breadth of the recognised epitopes constitute *per se *valid correlates of protection [20-22]. Recently, studies have shown that polyfunctional CD8 T-cell responses are preferentially observed in long term non-progressors (LTNP) when compared to persons with progressive disease [23]. Furthermore, antigen-specific terminally differentiated CD8 T-cells, defined by the lineage markers CCR7 and CD45RA, have been preferentially found in long-term non-progressors [24] and early infections with future control of HIV-1 viremia [25]. These findings highlight the importance of developing assays able to simultaneously measure several parameters in the same sample and strongly suggest the use of flow cytometry to monitor immune responses.

In this regard, we have developed a 9-colour ICS that allows the simultaneous determination of the function and the memory phenotype of antigen specific CD4 and CD8 T-cells. The assay has the capacity to detect the cytokines IFN-γ and IL-2, the chemokine MIP-1β and the activation marker CD154. For the characterization of the memory phenotype, we used CD45RA, an isoform of a membrane phosphatase that is expressed by both naïve and terminally differentiated T-cells [26]. Here, we compared the sensitivity of our recently established 9-colour ICS with ELISPOT assays performed in two different experienced laboratories. In our experimental setting, taking advantage of the simultaneous detection of IFN-γ and MIP-1β producing T-cells, we demonstrated a similar or superior capacity of our ICS assay to detect low magnitude IFN-γ-mediated responses.

## Results

### The simultaneous evaluation of IFN-γ+ MIP-1β+ T-cells increases the capacity to detect IFN-γ responses in ICS

**Figure 1:**
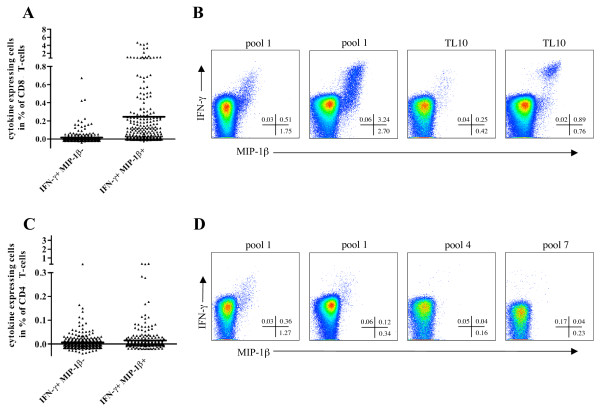
**IFN-γ and MIP1-β expression in CD8 and CD4 T-cells stimulated with HIV-1-derived antigens**. Percentages of IFN-γ+ MIP-1β- and IFN-γ+ MIP-1β+ CD8 (A) or CD4 (C) T-cells are shown for a total of 275 samples. The mean is depicted for each T-cell population. Representative pseudo-colour dot plots of data gated on living CD8+ CD3+ lymphocytes (B) or living CD4+ CD3+ lymphocytes (D) from 4 different patients are shown. In each plot the percentage of IFN-γ+ MIP-1β-, IFN-γ+ MIP-1β+ and IFN-γ- MIP-1β+ is indicated in the bottom-right corner. The pools used for PBMC stimulation are described in Table 1. TL10, TPGPGVRYPL.

**Figure 2:**
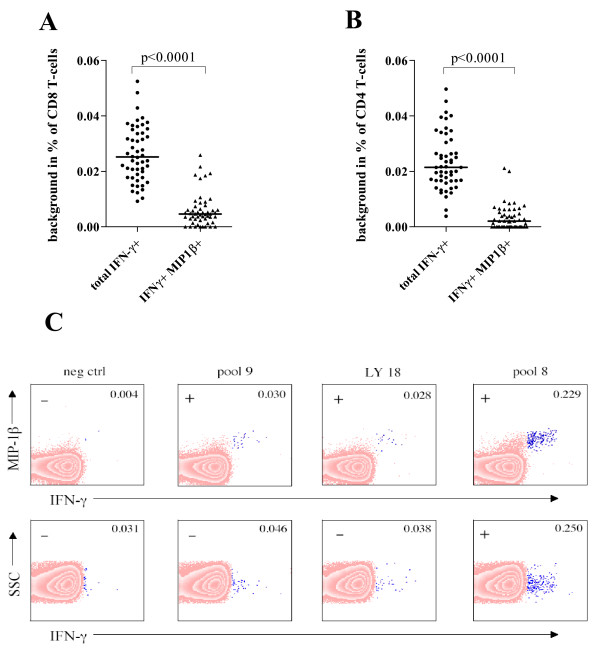
**Magnitude of IFN-γ+ MIP-1β+ T-cells and IFN-γ+ T-cells in mock stimulated samples**. Percentages of total IFN-γ+ and IFN-γ+ MIP-1β+ CD8 (A) or CD4 (B) T-cells are shown. The lines indicate the median percentage of the observed background. P values were determined by Wilcoxon matched pairs test. In (C) representative data from one study subject are shown. PBMC are gated on CD8+ CD3+ lymphocytes and were stimulated as indicated at the top of the figure. The peptide LDLWIYHTQGYFPDWQNY (LY18), included in pool 8, was here used alone. Data were analyzed with the IFN-γ+ MIP-1β+ (upper row) or the total IFN-γ+ (bottom row) data analysis system. The percentage of IFN-γ+ MIP-1β+ and total IFN-γ+ CD8 T-cells is indicated in the upper-right corner of each plot. Samples were scored as positive or negative (upper-left corner) according to the following procedure. After background subtraction, the 90 percentile of the negative values was calculated and this value was considered as a threshold. Samples were considered positive when higher than the threshold and at least 2 times higher than their respective mock stimulated control.

The 9 colour ICS assay established in our laboratory is routinely used to measure HIV-1 specific immune responses in different clinical settings. The cumulative analysis of 275 samples obtained from 31 HIV-1 positive individuals stimulated with peptides derived from 5 different HIV-1 proteins (Table 1) revealed an interesting feature of IFN-γ-based responses. Upon antigenic stimulation the majority of the IFN-γ producing CD8 T-cells were also producing MIP-1β (IFN-γ+ MIP-1β+ CD8 T-cells in %: mean ± SD, 0.245 ± 0.6341), whereas CD8 T-cells characterized by the sole production of IFN-γ were rarely detected (IFN-γ+ MIP-1β- CD8 T-cells in %: mean ± SD, 0.016 ± 0.0652) (Figure 1A and 1B). This trend was observed for all the CD8 T-cell responses whereas the few detected CD4 T-cell responses were more heterogeneous, since antigen-specific cells producing IFN-γ but not MIP-1β were detectable (IFN-γ+ MIP-1β+ CD4 T-cells in %: mean ± SD, 0.014 ± 0.0500; IFN-γ+ MIP-1β- CD4 T-cells in %: mean ± SD, 0.006 ± 0.0328; Figure 1C and 1D). The analysis of T-cells positive for both markers is of particular interest, since the simultaneous evaluation of two functions is supposed to decrease the non-specific background [23]. In order to investigate this observation in our experimental setting, we analyzed 52 mock stimulated samples from 31 HIV-1 positive subjects. Mock stimulated samples were run for each analyzed patient to measure spontaneous cytokine production and unspecific antibody staining. They were processed as the other samples but in the absence of antigenic peptides. The measured background was significantly reduced (around 4-fold lower; p < 0.0001, Wilcoxon matched pairs test) in the IFN-γ+ MIP-1β+ CD8 T-cells when compared to the total IFN-γ+ CD8 T-cells (Figure 2A). Similarly, we observed a 7-fold decrease (p < 0.0001, Wilcoxon matched pairs test) of the non-specific background in IFN-γ+ MIP-1β+ CD4 T-cells when compared to total IFN-γ+ CD4 T-cells (Figure 2B). Representative plots of responding CD8 T-cells and their negative control are shown in Figure 2C.

**Table 1:** Characteristics of the peptide pools used in this study

Pool	Antigen	HIV-1 subtype	Length (aa)	Overlap (aa)	# of peptides
1	Nef	LAI	20	10	20
2	Tat	LAI	20	10	8
3	Rev	LAI	20	10	11
4	p24	LAI	20	10	22
5	p17	SF2	15	5	13
6	Nef	LAI	8–11	NA	16
7	Nef (1–96)	Bru	variable	variable	15
8	Nef (95–205)	Bru	variable	variable	15
9	Tat	BH10	variable	variable	11

aa = Amino acids; NA = not applicable

**Figure 3:**
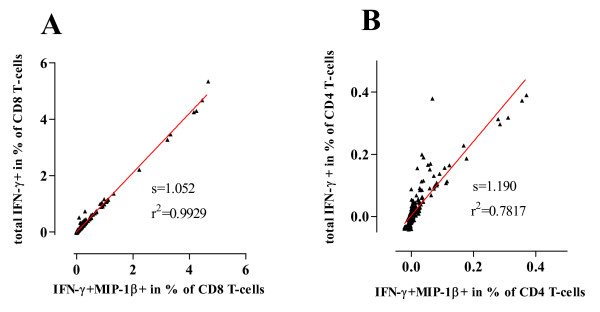
**Linear regression analysis**. Linear regression analysis between frequencies of IFN-γ+ MIP-1β+ T-cells and total IFN-γ+ T-cells is shown for CD8 (A) and CD4 (B) T-cells. The slope (s) and the goodness of fit (r^2^) are indicated in each graph. The regression line is depicted in each graph.

A linear regression analysis was performed to examine the correlation between percentages of total IFN-γ+ and percentages of IFN-γ+ MIP-1β+ CD8 and CD4 T-cells in samples stimulated with HIV-1-derived peptides. Percentages of total IFN-γ+ and IFN-γ+ MIP-1β+ CD8 T-cells showed a goodness of fit of r^2 ^= 0.9929 and a slope of 1.052 demonstrating an almost perfect linearity of the two measurements (Figure 3A). The goodness of fit was slightly lower for CD4 T-cells; although it was still characterized by an r^2 ^value of 0.7817 (Figure 3B). The slope was 1.190, confirming the presence of HIV-1 specific CD4 T-cells producing IFN-γ but not MIP-1β, as previously shown (Figure 1C and 1D).

Since the numbers of IFN-γ+ MIP-1β+ T-cells were essentially equivalent to those of total IFN-γ+ T-cells whereas the background was strongly decreased in the former, we made the assumption that the evaluation of double positive IFN-γ+ MIP-1β+ T-cells could represent an interesting option to increase the sensitivity of the ICS assay in the detection of IFN-γ mediated HIV-1-specific responses.

**Figure 4:**
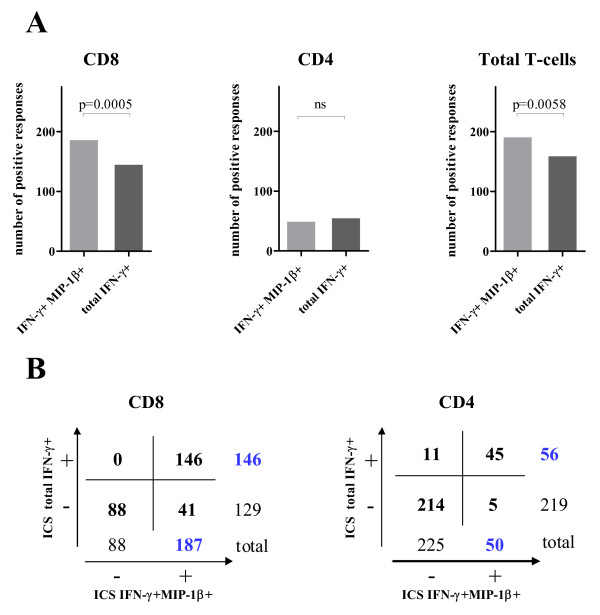
**Number of detected positive responses**. (A) The histogram plots show the number of positive CD8, CD4 or total T-cell responses detected with the IFN-γ+ MIP-1β+ and the total IFN-γ+ data evaluation systems. The p values (Fisher's exact test) are shown for each graph. Not significant difference (ns). (B) 2 × 2 contingency tables comparing the two data evaluation systems are shown for CD8 and CD4 T-cell responses.

In order to compare the sensitivity of the two modalities to evaluate the IFN-γ T-cell response, we analyzed the previously described 275 independent samples (Figure 4A). We calculated the 90^th ^percentile of the negative values after background subtraction and this value was considered as a threshold. Samples were considered positive when higher than the threshold and at least 2-fold higher than their respective negative control. In the CD8 T-cell population, 187 positive responses were detected using the IFN-γ+ MIP-1β+ data evaluation, while only 146 positive responses were detected using the total IFN-γ+ data evaluation. The difference was significant performing a Fisher's exact test (p = 0.0005). The difference between positive CD4 T-cell responses calculated using the two modalities was not significant. When CD8 and CD4 T-cell responses were considered together, the difference achieved significance with a p value of 0.0058 (Fisher's exact test). The contingency tables in Figure 4B show that the IFN-γ+ MIP-1β+ data evaluation allowed the detection of 41 CD8 responses that were otherwise missed by evaluation of the total IFN-γ+ T-cells. As expected, the simultaneous detection of IFN-γ+ and MIP-1β+ did not increase the capacity to detect antigen-specific CD4 T-cell responses. In fact, 11 CD4 responses were exclusively detected by the total IFN-γ+ data evaluation whereas 5 were exclusively observed with the simultaneous detection of IFN-γ+ and MIP-1β+.

### Evaluation of IFN-γ+ MIP1β+ cells increases the sensitivity of the ICS in comparison to the ELISPOT

ICS is generally considered less sensitive than ELISPOT in detecting low magnitude responses [8-10]. Therefore, we tested whether the simultaneous detection of MIP-1β and IFN-γ might increase its sensitivity in comparison to two ELISPOT assays performed in independent laboratories. Each laboratory used its own ELISPOT method, including a different ELISPOT reader and a different procedure to determine positive responses (see Methods). To facilitate the comparison with the ELISPOT, ICS results were expressed as the sum of the CD8 and CD4 responses and a response in ICS was considered positive when a CD8 or a CD4 response was scored as positive.

**Figure 5:**
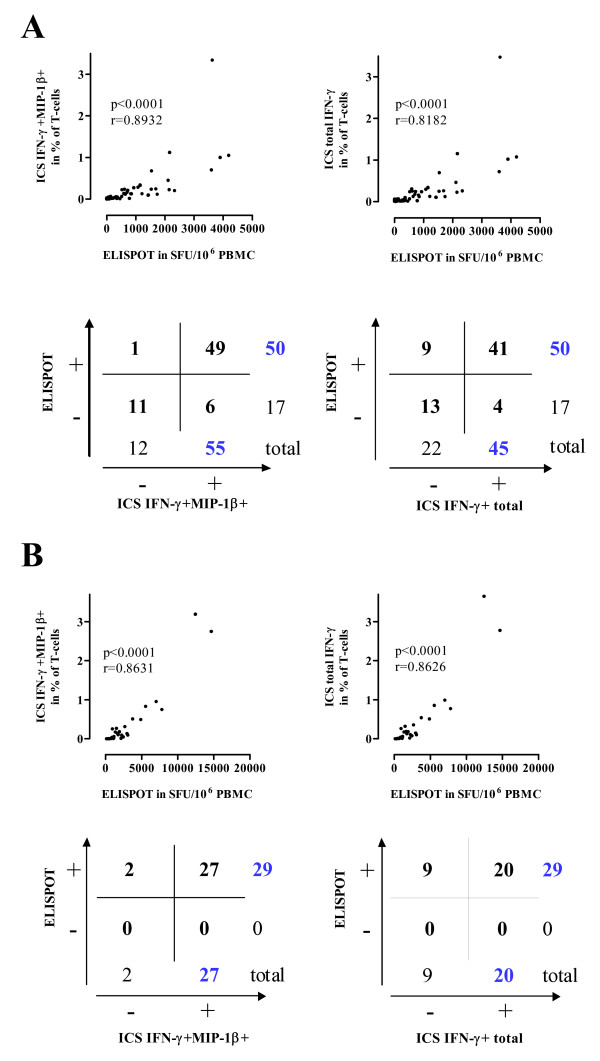
**Comparison between the ICS and two independently performed ELISPOT assays**. The two ICS data evaluation systems are compared with ELISPOT assays performed by laboratory 1 (A) and laboratory 2 (B). Correlations between frequencies of responding T-cells detected by ELISPOT and by ICS using the IFN-γ+ MIP-1β+ or the total IFN-γ+ data evaluation system are determined by Spearman's rank correlation. r and p values are shown in each graph. 2 × 2 contingency tables comparing the positive T-cell responses detected by ELISPOT and by ICS with the two data evaluation systems are also shown.

Laboratory 1 analyzed 67 samples from 17 HIV-1 infected subjects stimulated with 14 different peptide formulations derived from two different HIV-1 proteins. Correlation analysis of the responses measured by ELISPOT and by ICS expressed in terms of IFN-γ+ MIP-1β+ CD8 T-cells or total IFN-γ+ CD8 T-cells demonstrated in both cases a significant correlation (Figure 5A). The ELISPOT detected 50 positive responses in 67 samples, while the ICS positive responses expressed as IFN-γ+ MIP-1β+ CD8 or CD4 T-cells were 55 and the ICS positive responses expressed as total IFN-γ+ CD8 or CD4 T-cells were 45. By measuring IFN-γ+ MIP-1β+ we detected 6 positive responses that were otherwise missed by ELISPOT whereas, in contrast, only 1 response detected by ELISPOT was missed in our ICS determination. By determination of the total IFN-γ+ cells, we were able to detect 4 positive responses that were missed by the ELIPOT, but the ELISPOT was able to detect 9 responses missed by the ICS. Of note, 8 out of 10 positive responses that were additionally detected using the IFN-γ+ MIP-1β+ data evaluation were also scored positive using ELISPOT demonstrating an improvement of the concordance between the assays.

Laboratory 2 analyzed 29 samples obtained from 3 HIV-1 infected subjects stimulated with 18 different peptide formulations derived from 5 HIV-1 proteins using an ELISPOT assay approved by the Cancer Vaccine Consortium [27]. As observed in the results generated from the first laboratory, the correlation with the ELISPOT results was significant for both ICS methodologies (Figure 5B). A total of 29 positive responses were detected by ELISPOT, while 27 and 20 positive responses were detected by ICS using the IFN-γ+ MIP-1β+ and the total IFN-γ+ methods, respectively. Only 2 positive responses were lost by the IFN-γ+ MIP-1β+ data evaluation system, whereas 9 responses were lost by the total IFN-γ+ data evaluation system in comparison to the ELISPOT performed in laboratory 2. These combined results of the 2 laboratories demonstrated that the new evaluation method based on the simultaneous detection of IFN-γ and MIP-1β increased the capacity of the ICS to detect low-magnitude responses.

### Influence of the variation in cell number input in the ICS assay

**Figure 6:**
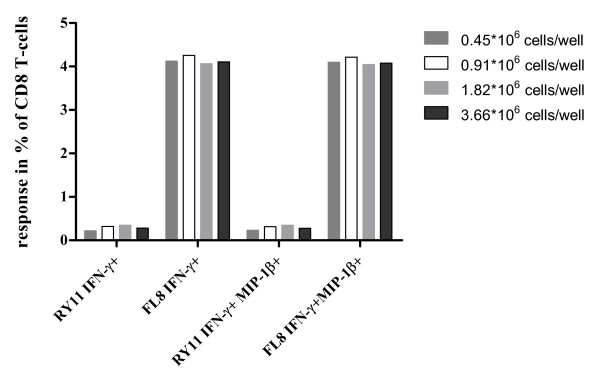
**Variation in the number of cells/well in ICS assay**. Different amounts of PBMC were stimulated with 2 different Nef derived optimal CD8 epitopes (FLKEKGGL, FL8 and RRQDILDLWIY, RY11). Analyzed responses are shown on the x axis.

Cell counting is a basic technique in use in all cell culture laboratories. Nevertheless, it constitutes an important source of experimental error [27]. The number of cells per sample is a critical parameter in the ELISPOT assay, since results are directly calculated from the total amount of cells seeded in each well. In contrast, in the ICS assay responding cells are calculated as a percentage of CD4 or CD8 T-cells and therefore the results are independent from the total number of cells used in each experimental sample. However, variation in the cell number might still affect the experimental outcome because of changes in the proportion between the amount of cells, growth factors and stimulants. Therefore, we tested the impact of varying the amount of PBMC per experimental sample in our 9-colour ICS assay. Stimulation with 2 different peptides representing optimal CD8 epitopes was performed using 0.45, 0.91, 1.82 and 3.66 million of cells/well, while the amount of peptides was kept constant at 2 μg/ml. There was neither a trend nor a high variation between the results for either the total IFN-γ+ response as well as for the combined IFN-γ and MIP-1β positive cells (Figure 6). Of note, the background levels were not affected by the number of cells seeded per well.

## Discussion

In the present study, we provide experimental evidence in support of a combined 9-colour IFN-γ and MIP-1β ICS method that, unlike commonly used methods based on the flow-cytometric detection of IFN-γ, achieves sensitivity comparable to that typical of ELISPOT assays.

ELISPOT and ICS assays are widely used to measure specific immune responses in different experimental settings. IFN-γ ELISPOT is considered the gold standard for the evaluation of the immune response in vaccination trials even though accumulating evidence demonstrates that the measurement of a single immunological marker does not provide sufficient information about the efficacy of a specific immune response [9,28,29]. In addition, recent disappointing results of phase III efficacy HIV-1 vaccination trials [30] underscored the need for a better evaluation of the immune response in phase I and II clinical trials. A supporting issue in favour of the use of the IFN-γ ELISPOT as primary assay in vaccine trials is its supposed higher sensitivity in comparison to other immune monitoring assays such as ICS [8-10,12]. Here, we demonstrated that the use of a combined detection of IFN-γ and MIP-1β could scale-up the sensitivity of ICS assays to levels comparable to those of IFN-γ-based ELISPOT. In this regard, the key observation is that the majority of the IFN-γ producing T-cells are simultaneously producing MIP-1β rendering this new modality of evaluation equivalent to the measurement of the total IFN-γ producing T-cells with the relevant advantage of a consistent decrease of the background that in turn increases the sensitivity of the assay.

It is unlikely that the increased sensitivity of the IFN-γ+ MIP-1β+ data evaluation is due to false positive detections, since simultaneous unspecific binding of two antibodies to the same cell is less probable than unspecific binding of a single antibody. In addition, the majority of samples scored as positive with the IFN-γ+ MIP-1β+ data evaluation were scored as positive using the ELISPOT as well, indicating that the increased sensitivity was not due to a higher number of false positive detections but due to a better capacity of the IFN-γ+ MIP-1β+ data evaluation to discriminate positive responses in comparison to the total IFN-γ+ data evaluation.

Our results provide support for an expanded use of polychromatic flow cytometry as primary assay in vaccine trials. The ICS method optimized in our laboratory allows the simultaneous measurement of several fluorescence markers without losing sensitivity in comparison to the gold standard IFN-γ ELISPOT. In our setting, we used a 9 colour ICS; however, the same method can be applied to any staining combination including IFN-γ and MIP-1β in combination with the appropriate lineage markers. Thus, for investigators with no access to sophisticated flow cytometers, a simplified panel can be used for immune-monitoring purpose as alternative to the ELISPOT not losing sensitivity and with the advantage to discriminate CD4 and CD8 mediated responses. In alternative, more complex staining combinations could be designed for laboratory facilities where complex instrumentation is available, provided the inclusion of the simultaneous measurement of IFN-γ and MIP-1β.

Our present study was limited to the analysis of the HIV-1-specific T-cell responses. Nevertheless, this method can be extended to other specific immune responses if T-cells expressing IFN-γ and MIP-1β represent the majority of the total IFN-γ producing T-cells. In this regard, a possible extension of our methodology is the coupling of an activation marker (i.e. CD69, CD154, etc.) to the measurement of cytokines or chemokines (i.e, IFN-γ, IL-2 and MIP-1β). As a general rule, targeting 2 or more molecules on the same cell population should increase the sensitivity of the assay for the selected cell population. Since flow cytometry has been recently advanced by the development of new instrumentation and reagents, the inclusion of more markers in a single sample should aim not only to increase the amount of information per cell but also to increase the sensitivity for populations of special interest.

Finally, in the present study we demonstrate that the number of cells used in each sample does not affect the readout of the ICS. Since the procedure of manual cell counting is a usual source of experimental error and the number of cells directly affects the ELISPOT readout, our data support the concept of a reduced experimental error associated with the use of ICS assays and strengthens the idea to apply ICS as primary assay in vaccine trials.

## Conclusion

The simultaneous detection of IFN-γ and MIP-1β provides a clear advantage for the detection of low HIV-1 specific responses compared to the classical way to analyze the total IFN-γ producing T-cells by ICS. The comparison with the results generated by ELISPOT independently by two experienced laboratories demonstrates that the combined IFN-γ+ MIP-1β+ evaluation system allows for the detection of low HIV-1 specific IFN-γ responses to a similar or even higher extent, as they can be detected using ELISPOT assays. The application of the IFN-γ+ MIP-1β+ method in other diseases and immunological fields remains to be assessed. These findings are important to guide the choice for suitable immune assays and to build reagent panels able to accurately characterize the phenotype and function of responding T-cells in a highly sensitive way.

## Methods

### Study samples

PBMC obtained from 31 HIV-1 infected individuals were analyzed in the present study. Their median CD4 T-cell count was 502 cells/μl (range 229 to 1,042). Twenty-one study subjects were under antiretroviral therapy and 16 of them had undetectable viral load. When detectable the median viral load was 2,581 RNA copies/ml (range 151 to 50,577). Six of the study subjects under antiretroviral therapy with undetectable (< 50 copies of RNA/ml) viral load underwent treatment interruption and their range of viremia was then from 600 to 49,600 RNA copies/ml at the time of sampling.

### Peptides

As shown in Table 1, nine different HIV-1 derived peptide pools were used to stimulate PBMC: (1) 20-mer peptides overlapping by 10 amino acids spanning the HIV-1 LAI Nef protein; (2) 20-mer peptides overlapping by 10 amino acids spanning the HIV-1 LAI Tat protein; (3) 20-mer peptides overlapping by 10 amino acids spanning the HIV-1 LAI Rev protein; (4) 20-mer peptides overlapping by 10 amino acids spanning the HIV-1 LAI p24 protein; (5) 15-mer peptides overlapping by 5 amino acids spanning the HIV-1 SF2 p17 protein; (6) pool of 16 Nef derived peptides corresponding to previously described optimal CD8 epitopes [31]; (7) variable length overlapping peptides spanning the 1 to 96 region of HIV-1 Bru Nef; (8) variable length overlapping peptides spanning the 96 to 205 region of HIV-1 Bru Nef and (9) variable length overlapping peptides spanning HIV-1 BH10 Tat. Pool 1 to 6 and 7 to 9 were previously described by Cosma et al [32] and Vardas et al. [33], respectively. Several peptides contained in the pools 7, 8 and 9 were used alone in some experiments. The following peptides corresponding to previously described optimal CD8 epitopes [31] were also used in some stimulation experiments: FLKEKGGL (FL8), TPGPGVRYPL (TL10), YPLTFGWCY and RRQDILDLWIY (RY11). All the peptide pools were tested for specificity in healthy subjects in previous studies [32,33].

### Intracellular cytokine staining

Cryopreserved PBMC were used for the ICS assay. After thawing, 10^6 ^PBMC were resuspended in 150 μl RPMI 1640 (Cambrex, Taufkirchen, Germany) supplemented with 10% FCS. The stimulation was performed with 0.4 μg peptide/10^6 ^cells in the presence of 1.3 μg/ml anti CD28 and 1.3 μg/ml anti CD49d costimulatory antibodies (Becton Dickinson, Heidelberg, Germany). Following 60 min incubation, 10 μg/ml of Brefeldin A (Sigma-Aldrich, Taufkirchen, Germany) were added to the cell suspension and the incubation carried out for additional 4 h. Stimulated cells were then resuspended in Stain Buffer (0,2% BSA, 0,09% Na Azide in DPBS; Becton Dickinson) and incubated with the photoreactive fluorescent label ethidium monoazide (EMA; Molecular Probes/Invitrogen, Karlsruhe, Germany) to asses their viability. After washing, cells were fixed and permeabilized using the BD Cytofix/Cytoperm™ Kit (Becton Dickinson). Then, the following fluorochrome-conjugated antibodies were added: CD8-PacB (DAKO cytomation, Hamburg, Germany), CD3-AmCyan, CD4-PerCP, CD45RA-PECy7, CD154-FITC, IFN-γ-Al700, IL-2-APC and MIP1β-PE (Becton Dickinson). Incubation was carried out on ice for 30 min and after washing, cells were acquired using an LSRII flow cytometer (Becton Dickinson) equipped with a high throughput system. Sample analysis was performed using FlowJo version 8.5.3 (Tree Star, Ashland, OR). The gating strategy is shown in Additional file 1. Lymphocytes were gated on a forward scatter area versus side scatter area pseudo-colour dot plot and dead cells were removed according to EMA staining. CD3+ events were gated versus IFN-γ, IL-2, MIP-1β and CD154 to account for down-regulation. CD3+ events were then combined together using the Boolean operator "Or". The same procedure was used to subsequently gate CD8+ events. CD4+ events were excluded before creating a gate for each function or phenotype. After background subtraction, the 90 percentile of the negative values was calculated and this value was considered as a threshold. Samples were considered positive when higher than the threshold and at least 2 times higher than their respective mock stimulated control.

### ELISPOT assay (laboratory 1)

Laboratory 1 used the TriSpot™ Human IFN-γ/IL-2 ELISPOT Kit (Endogen, Rockford, IL/USA) according to the manufacturer instructions. Briefly, PBMC from ACD whole blood were separated on Lymphoprep™ (Axis-Shield PoC, Oslo, Norway), washed in RPMI medium (RPMI 1640, supplemented with 100 U/ml penicillin, 100 μg/ml streptomycin and 2 mM L-glutamine, all from BioWhittaker Europe, Verviers, Belgium) and counted by Trypan Blue exclusion for assessing viability. After resuspension in complete medium (RPMI medium supplemented with 10% heat inactivated fetal bovine serum, BioWhittaker), PBMC were transferred to the ELISPOT plate with a concentration of 0.8 to 2 × 10^5^cells/well in duplicate. Peptides were added at a final concentration of 3 μg/ml each. PBMC in medium alone or stimulated with phytohemagglutinin (PHA-P, Sigma) at 5 μg/ml were used as negative and positive controls, respectively. Incubation was carried out at 37°C in a 5% CO_2 _incubator for 18 hours. The resulting spots were counted using the Automated ELISA-Spot Assay Video Analysis System Eli-Scan with the software Eli.Analyse V4.2 (A.EL.VIS, Hannover, Germany). PBMC from each study subject were mock stimulated in duplicate and the mean background value subtracted from the mean of the duplicate samples. Responses were empirically scored as positive when the stimulated sample minus background value was > 50 SFU per 10^6 ^PBMC and higher than the mean value of the negative controls plus 2 standard deviations. Only spots positive for IFN-γ production were taken in consideration for the present study.

### ELISPOT assay (laboratory 2)

Frozen PBMC were thawed, washed with CTL Wash™ Supplement culture medium (Cellular Technology Ltd., Cleveland, Ohio) plus benzonase nuclease (50 U/ml; Novagen, Madison, WI), rested for 3 h at 37°C, counted and seeded at 1 to 2 × 10^5 ^cells in triplicates on antibody precoated PVDF plates (Mabtech AB, Nacka, Sweden). The capture antibody (Mabtech) was the IFN-γ-specific clone 1-D1K. Beforehand, the plates were incubated at 37°C in RPMI 1640 culture medium supplemented with 2 mM L-glutamine, 1 mM sodium pyruvate, penicillin/streptomycin (100 U/ml) and 10% human AB serum (BioWhittaker, Verviers, Belgium) to block unspecific binding. The PBMC were stimulated directly with different peptides and peptide pools (2 μg/ml), and assessed in the ELISPOT assay after 24 h of culture in CTL Test™ medium. The development of the spots was performed as described previously [34] with the following exceptions: the plates were extensively washed first with PBS/0.05% Tween20, then with only PBS, incubated with a directly streptavidin-alkaline phosphatase (ALP) conjugated biotinylated detection antibody clone 7-B6-1 (Mabtech), washed again and a ready-to-use BCIP/NBT-plus substrate solution was used (Mabtech). Spots were counted using the AID reader system ELR03 with the software version 4.0 (AID Autoimmun Diagnostika GmbH, Strassberg, Germany). Responses were scored as positive if the test wells contained a mean number of spot-forming units (SFU) higher than the mean value plus 2 standard deviations in negative control wells. The present ELISPOT standard operation procedure was approved by the international panel analysis of the Cancer Vaccine Consortium [27].

### Statistical analysis

All statistical tests were performed with PRISM^® ^5.01 (GraphPad Software Inc., San Diego, CA). The significance level was 0.05 for all statistical tests.

## Competing interests

The authors declare that they have no competing interests.

## Authors' contributions

SK and AC conceived the study. SK, CJD, SA, SH and BS performed experiments. SN, GT, JRB, HJS, FDG, MT and GP participated to the collection of patient samples. SK, CJD, SA, SH, BS, PB, HP, MM and AC analyzed data. PL and VE contributed to research and critical discussion. SK and AC wrote the paper. All authors provided editorial comments and assistance.

## Supplementary Material

Additional file 1 **Gating strategy**. Representative example showing the gating strategy of the 9 colour ICS applied on a PBMC sample stimulated with peptide pool 6. Lymphocytes are gated on a forward scatter area (FSC-A) versus side scatter area (SSC-A) pseudo-colour dot plot (A) and dead cells are removed according to EMA staining (B). CD3+ events are gated versus CD154 (C), IFN-γ (D), IL-2 (E) and MIP-1β (F) to account for down-regulation. CD3+ events are then combined together using the Boolean operator "Or". The same procedure is used to subsequently gate CD8+ (G, H, I and J) and CD4+ (K, L, M and N) events. CD4+ events are excluded from the CD8+ population using the exclusion gate in O before creating a gate for each function or phenotype (P, Q, R, S and T). CD8+ events are excluded from the CD4+ population using the exclusion gate in O before creating a gate for each function or phenotype (U, V, W, X and Y). Click here for file
