# Supplementary figures and images for: The intracellular detection of MIP-1beta enhances the capacity to detect IFN-gamma mediated HIV-1-specific CD8 T-cell responses in a flow cytometric setting providing a sensitive alternative to the ELISPOT
Source: AIDS Res Ther. 2008 Oct 6;5:22. doi: 10.1186/1742-6405-5-22 (PMC2569082; doi:10.1186/1742-6405-5-22)

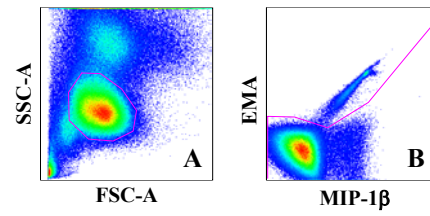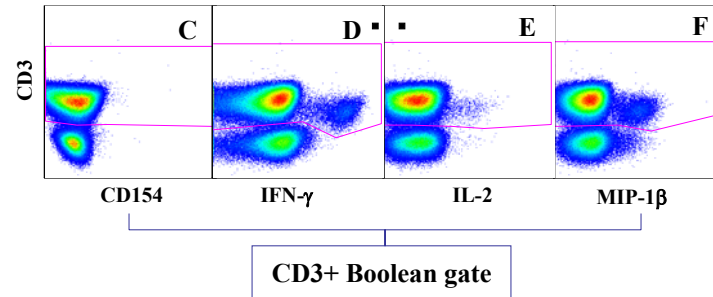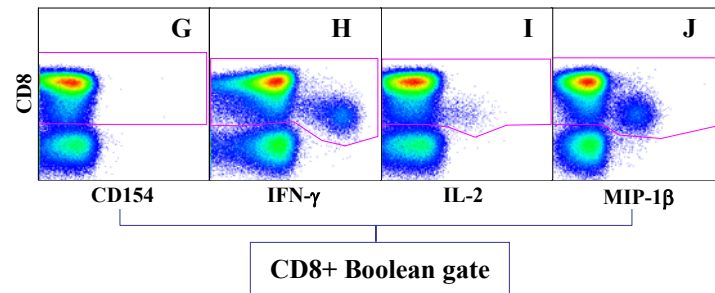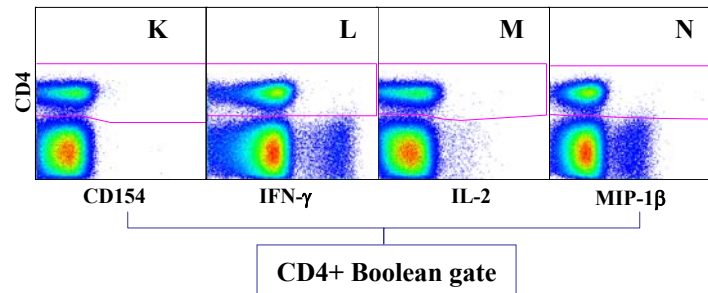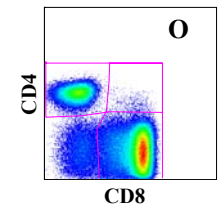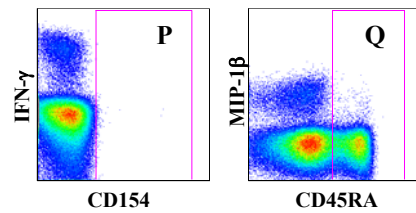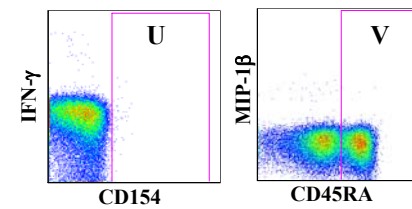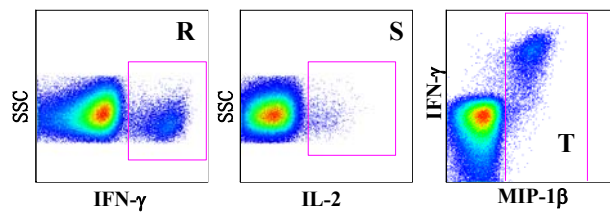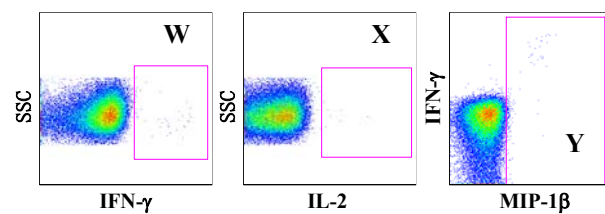

Supplement: Additional file 1 — Gating strategy. Representative example showing the gating strategy of the 9 colour ICS applied on a PBMC sample stimulated with peptide pool 6. Lymphocytes are gated on a forward scatter area (FSC-A) versus side scatter area (SSC-A) pseudo-colour dot plot (A) and dead cells are removed according to EMA staining (B). CD3+ events are gated versus CD154 (C), IFN-γ (D), IL-2 (E) and MIP-1β (F) to account for down-regulation. CD3+ events are then combined together using the Boolean operator "Or". The same procedure is used to subsequently gate CD8+ (G, H, I and J) and CD4+ (K, L, M and N) events. CD4+ events are excluded from the CD8+ population using the exclusion gate in O before creating a gate for each function or phenotype (P, Q, R, S and T). CD8+ events are excluded from the CD4+ population using the exclusion gate in O before creating a gate for each function or phenotype (U, V, W, X and Y). [file 1742-6405-5-22-S1.pdf]
